# Predictors of pathological upstaging to pT3a after nephrectomy for cT1 renal tumors: a systematic review and meta-analysis

**DOI:** 10.3389/fonc.2026.1783597

**Published:** 2026-03-31

**Authors:** Yushi Ye, Junjie Bai, Yuzhong Ye, Bijuan Lin, Gaoyu Zou, Jianjia Huang, Chuye Zheng, Qianyi Qiu, Dongliang Zhong, Jun Lin, Jianhui Chen, Weizhong Cai, Shaoxing Zhu

**Affiliations:** 1 Department of Urology, Fujian Medical University Union Hospital, Fuzhou, China; 2 The Graduate School of Fujian Medical University, Fuzhou, China; 3 Shengli Clinical College of Fujian Medical University, Fuzhou, China; 4 Department of Urology, Fuzhou University Affiliated Provincial Hospital, Fuzhou, Fujian, China; 5 Department of Pharmacy, Fujian Medical University Union Hospital, Fuzhou, China

**Keywords:** meta-analysis, neoplasm staging, nephrectomy, renal cell carcinoma, risk factors

## Abstract

**Background:**

The potential predictors and clinical implications of postoperative pathological upstaging in patients with clinical stage T1 (cT1) renal cell carcinoma (RCC) remain insufficiently characterized.

**Methods:**

This systematic review was performed in accordance with the PRISMA guidelines. Databases including PubMed, Web of Science and Embase databases were searched. A total of 17 studies (involving 24957 patients) were included, and relevant factors were analyzed from the perspectives of patients’ baseline characteristics, pathology, and imaging findings.

**Result:**

A total of 17 studies involving 24,957 patients were included. The overall incidence of pathological upstaging was 7.46%. Through combined qualitative and quantitative analysis, we identified the following independent risk associated factors for pathological upstaging to pT3a: male gender (OR: 1.067; 95% CI: 1.029–1.107;p=0.001), age (OR = 1.029; 95% CI: 1.02–1.038;p = 0.0001), BMI (OR:1.016;95% CI: 1.004–1.027;p=0.0001),cT1b (OR: 5.865; 95% CI: 3.711- 9.267; p = 0.0001),presence of clinical symptoms(OR:1.987; 95% CI: 1.439- 2.744; p = 0.0001), RENAL score= 7-9 (OR = 2.480; 95% CI: 1.516–4.058;p = 0.0001),necrosis on imaging(OR = 2.347;95% CI: 1.570–3.509;p = 0.0001), irregular margins (OR = 2.874; 95% CI: 1.760–4.693;p = 0.0001)and Hilus involvement (OR = 2.134;95%CI: 1.367-3.333;p = 0.001).Furthermore, the unadjusted meta-analysis results indicated an increased risk of recurrence in patients who upstaged to pT3a (HR = 3.50,95% CI = 2.00-6.13). After multivariable adjustment, the association remained consistent and in the same direction (HR = 2.60,95% CI = 1.86-3.64).

**Conclusion:**

Current evidence suggests that 9 variables, including advanced age, gender, and irregular margins may be predictors of postoperative pathological upstaging of cT1 RCC.

**Systematic review registration:**

https://www.crd.york.ac.uk/prospero/, identifier CRD420250640397.

## 1 Introduction

Renal cell carcinoma (RCC) accounts for approximately 3% of all cancers, with the highest incidence rates reported in Western countries (2). As the most common solid renal mass, RCC constitutes roughly 90% of all malignant renal tumors. The male-to-female incidence ratio of RCC is 1.5:1 (29). RCC comprises distinct subtypes characterized by specific histopathological and genetic features (19). In 2022, there were an estimated 434,840 new cases of RCC and 155,953 RCC-related deaths worldwide (2).

According to the European Association of Urology (EAU) guidelines on RCC, current treatment modalities for RCC include surgical resection, transarterial embolization, active surveillance, ablative therapy, neoadjuvant therapy, and postoperative adjuvant therapy and so on (2). As recommended in the guidelines, partial nephrectomy (PN) is the preferred treatment option for patients clinically diagnosed with stage T1 RCC (tumor size < 7 cm) (2, 4, 17), which offers advantages in preserving renal function, preventing chronic kidney disease (CKD), and reducing cardiovascular events (10). One study has shown that PN can lower the risk of new-onset CKD after surgery for localized renal tumors, particularly by preserving postoperative estimated glomerular filtration rate (eGFR) levels—specifically, reducing the incidence of eGFR < 60 mL/min/1.73 m² and eGFR < 45 mL/min/1.73 m² postoperatively (21). In contrast, radical nephrectomy (RN) has an advantage in reducing the risk of postoperative local recurrence (17).

Pathological upstaging refers to a clinical scenario in which preoperative imaging or biopsy indicates a low-grade or localized tumor, but postoperative pathology confirms the tumor to be of higher grade or greater biological aggressiveness. Studies have shown that the incidence of pathological upstaging after renal cancer surgery is 11%. For cT1 RCC patients, those who undergo PN exhibit a recurrence-free survival rate similar to those who undergo RN; however, cT1 RCC patients who are upstaged to pT3a after surgery have poorer oncological prognosis (3, 5). Compared with RN, PN is associated with a higher risk of pathological upstaging from clinical T1 to T3a in RCC patients, and these upstaged patients face an increased risk of both local recurrence and distant metastasis (18). Specifically, cT1 RCC patients who are upstaged to pT3a show worse survival outcomes than those without upstaging (9, 14). Therefore, pathological upstaging may affect the accuracy of staging, prognosis assessment, and subsequent treatment decision-making.

As more studies on postoperative recurrence in cT1 RCC have emerged in recent years, previous studies have certain limitations in terms of recency and comprehensiveness. Therefore, we conducted an updated systematic review of this issue, aiming to identify more accurate predictors and provide evidence for treatment selection in patients with cT1 RCC.

## 2 Methods

### 2.1 Literature search strategy

The work has been reported in accordance with AMSTAR (Assessing the methodological quality of systematic reviews) Guidelines (27). According to the Cochrane Collaboration recommendations and following the PRISMA Statement (22), we performed this review.

This systematic review was registered in PROSPERO, CRD420250640397. We conducted literature screening in PubMed, Web of Science and Embase databases, including articles published from the inception of each database up to August 2025. The review question was defined according to the PICO framework: patients with cT1 renal masses who underwent PN or RN (P), with pathological upstaging to pT3a (I) versus without upstaging (C), and outcomes including baseline characteristics, pathological features, survival outcomes, and predictors of upstaging (O).A combination of MeSH (Medical Subject Headings) terms and free-text terms was used for the search, with the search terms including “renal cell carcinoma”, “partial nephrectomy”, “radical nephrectomy”, “clinical T1”, “pathological T3” and so on. Two researchers independently assessed all included studies, and any discrepancies were resolved through discussion or consultation with a third researcher. Data extraction was performed independently by another researcher and documented in a standardized Microsoft Excel spreadsheet. For each included study, we recorded the study design, demographic and oncological characteristics of the subjects, patient prognosis, odds ratio (OR), hazard ratio (HR), 95% confidence interval (CI), and p-value.

### 2.2 Inclusion and exclusion criteria

#### 2.2.1 Inclusion criteria

Patients with cT1-stage RCC confirmed by histopathological examination;

Patients who underwent PN or RN;

Studies investigating the predictive factors for pathological upstaging to pT3a;

Studies with a literature quality score of ≥ 5 points assessed by the Newcastle-Ottawa Scale (NOS) (28).

#### 2.2.2 Exclusion criteria

Articles that do not provide information regarding pT1 and pT3 stages and did not conduct multivariate logistic regression analyses;

Studies identified as originating from the same research institution and/or author group, or those with overlapping data;

Non-original studies and duplicate publications.

### 2.3 Data extraction and quality evaluation

Data were independently extracted by two researchers and checked for accuracy, covering the following aspects: basic study information, patient baseline characteristics, imaging parameters, pathological features, and outcome measures.

The Newcastle-Ottawa Scale (NOS) was used to assess the methodological quality of the included studies across three dimensions: selection (4 points), comparability (2 points), and outcome assessment (3 points). Studies with a total score of ≥ 5 were classified as high-quality studies.

### 2.4 Calculation

In this study, risk factors associated with postoperative prognosis of cT1 RCC reported in two or more studies were included in the final data analysis. The odds ratios and their 95% CI derived from multivariable logistic regression analyses in the included articles were used for statistical pooling. For survival outcomes, hazard ratios were employed for calculation. The random-effects meta-analysis was conducted using an inverse-variance method. The pooled results were visually presented in the form of forest plots. Heterogeneity among studies was assessed using Cochran’s Q test and Higgins’ I² statistic. A P-value < 0.10 or an I² value > 50% for heterogeneity indicated the presence of significant heterogeneity. In cases of significant heterogeneity, a random-effects model was employed; otherwise, a fixed-effects model was applied. All statistical analyses were performed using Stata 18.

## 3 Result

**Figure 1:**
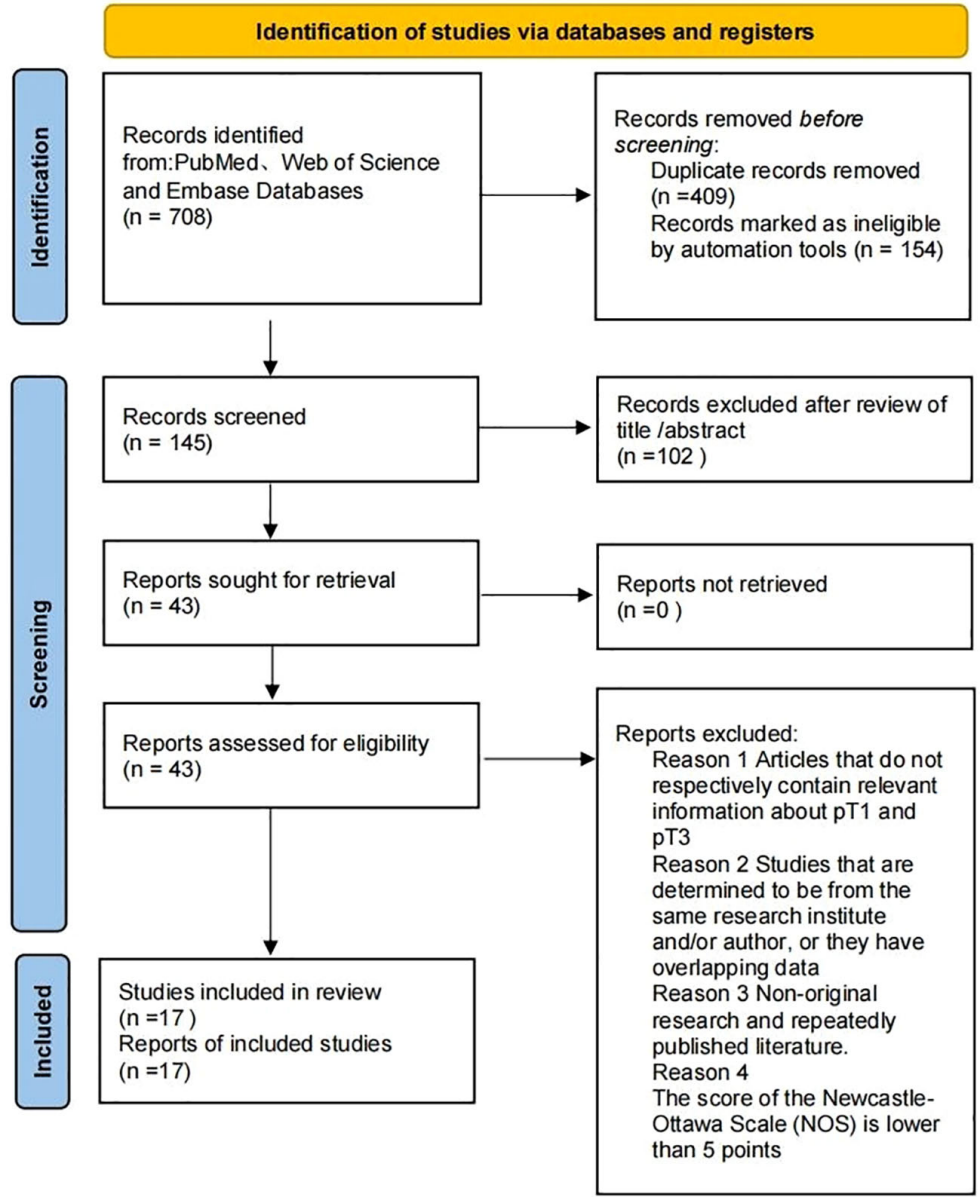
PRISMA flow chart.

A total of 708 references were retrieved from the pre-specified databases, including the PubMed, Web of Science and Embase databases. The methodological quality of the studies was assessed using the NOS, which assesses studies across three domains: selection, comparability, and outcome ascertainment. The total score was calculated as the sum of scores from these three domains (ranging from 0 to 9), with higher scores indicating better methodological quality. Studies with a total score of ≥5 were considered to be of sufficient quality for inclusion in the analysis. Discrepancies among researchers were resolved by consulting the senior author. Finally, 17 eligible articles were included (**Figure 1**).

A total of 24957 patients were included in the study, with pathologically upstaged cases accounting for 7.46% of the total study population. The study types comprised 15 retrospective cohort studies and 2 prospective cohort studies. Regarding quality assessment scores: 13 studies achieved 8 points on the Newcastle-Ottawa Scale (NOS), and 4 study scored 9 points. (**Tables 1**, **2**).

**Table 1:** The situation of the selected article.

Author	Year	Country	Total patients	RCC patients	Study design	pT3a	pT3a%	Treatment method	NOS score
Cao et al.	2022	China	510	510	Retrospective Cohort Study	102	20.00	RN&PN	9
Gorin et al.	2013	United States	1,096	855	Prospective Cohort Study	41	4.80	PN	9
Ishiyama et al	2024	Japan	1617	1617	Retrospective Cohort Study	28	1.73	PN	9
Lee et al	2018	South Korea	3431	3431	Retrospective Cohort Study	215	6.30	RN&PN	8
Jeong et al	2016	South Korea	987	987	Retrospective Cohort Study	91	9.02	RN&PN	8
Mei et al	2024	China	1012	1012	Retrospective Cohort Study	91	9.00	RN&PN	8
Mouracade et al	2017	United States	1042	1042	Retrospective Cohort Study	113	10.84	PN	8
Nayak et al	2016	Canada	2740	1513	Retrospective Cohort Study	134	9.25	RN&PN	8
Park et al	2024	South Korea	48	48	Prospective Cohort Study	12	25.00	PN	8
Pathak et al	2024	India	313	313	Retrospective Cohort Study	19	6.07	PN	8
Pruthi et al	2023	United States	6148	6148	Retrospective Cohort Study	681	11.08	RN&PN	9
Ramaswamy et al	2015	United States	494	494	Retrospective Cohort Study	66	13.36	RN&PN	8
Russell et al	2018	United States	1955	1955	Retrospective Cohort Study	95	4.86	PN	8
Sekito et al	2023	Japan	181	181	Retrospective Cohort Study	8	4.42	RN&PN	8
Teishima et al	2020	Japan	367	367	Retrospective Cohort Study	18	4.90	RN&PN	8
Veccia et al	2020	Italy	1640	1640	Retrospective Cohort Study	74	4.51	PN	8
Wang et al	2023	China	1376	1376	Retrospective Cohort Study	75	5.45	NSS&RN	8

RCC, renal cell carcinoma; NOS, Newcastle-Ottawa Scale; PN, partial nephrectom; RN, radical nephrectomy; NSS, nephron-sparing surgery.

**Table 2:** Predictive factors for the upgrade from cT1 to pT3.

Predictive factors	Number of studies	cT1/pT3a(%)	Tau^2	Chi^2	df	p value	I^2(%)	OR	95% CI	p value
Age	9	1205(8.89)	0.0001	14.02	9	0.122	35.8	1.029	1.02, 1.038	0.0001
Gender	8	840(7.35)	0.0251	13.21	7	0.067	47.0	1.067	1.029, 1.107	0.001
BMI	3	857(10.33)	0.00001	2.73	3	0.435	0.0	1.016	1.004,1.027	0.0001
cT1b	4	156(3.63)	0.0335	3.45	3	0.327	13.0	5.865	3.711,9.267	0.0001
Having Clinical System	4	285(5.71)	0.00001	0.87	3	0.832	0.0	1.987	1.439, 2.744	0.0001
Necrosis Suggested by Imaging	2	166(6.95)	0.00001	0.81	1	0.367	0.0	2.347	1.570,3.509	0.0001
Irregular Tumor Margin	3	184(6.68)	0.0674	3.10	2	0.212	35.5	2.874	1.760–4.693	0.0001
Hilus Involvement	4	224(5.78)	0.0549	4.06	3	0.255	26.2	2.134	1.367,3.333	0.001
RENAL Score(7-9)	2	154(8.12)	0.00001	0.22	1	0.642	0.0	2.480	1.516,4.058	0.0001

**Figure 2:**
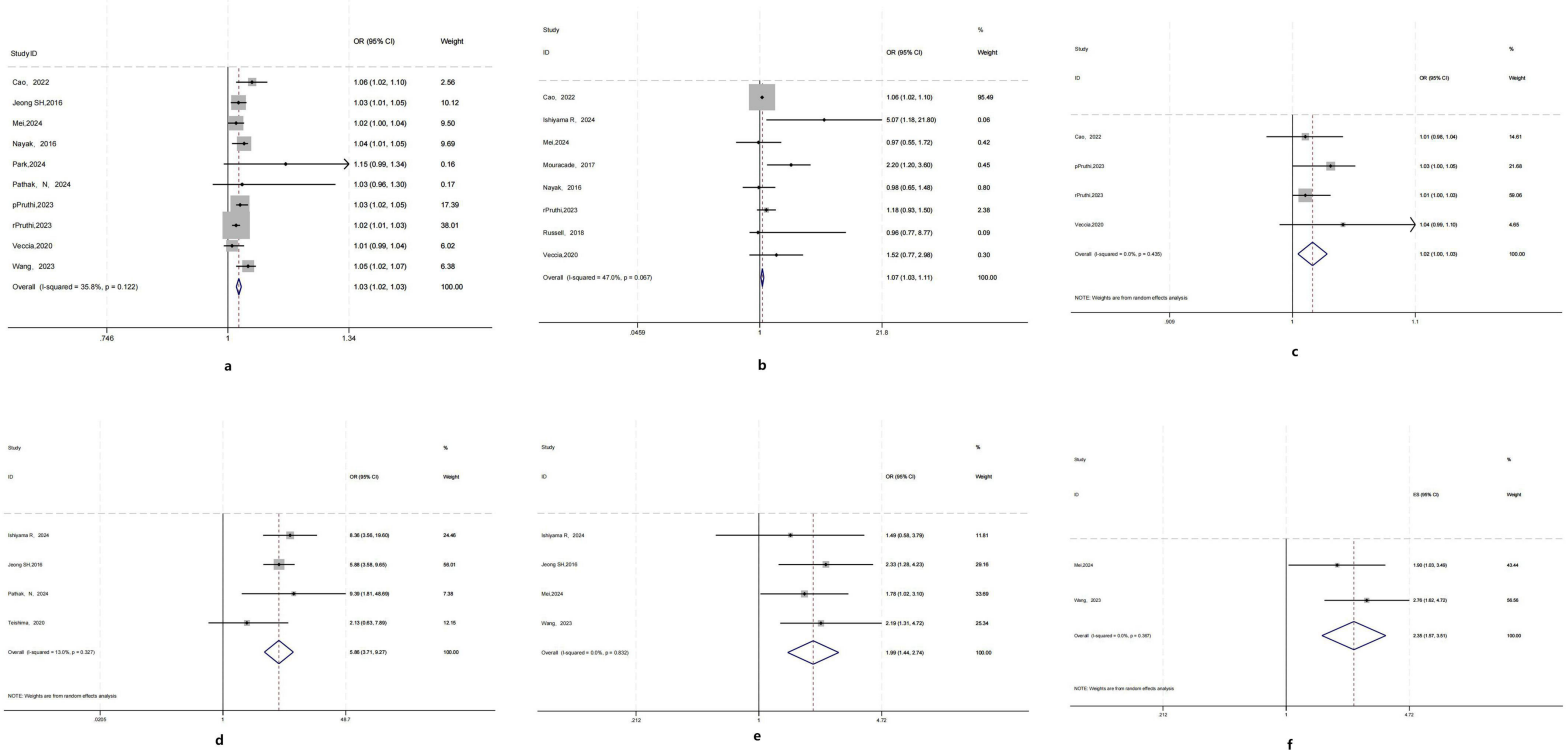
Forest plots of predictors of pathological upstaging. [**(a)** Age; **(b)** Male sex; **(c)** BMI; **(d)** Clinical T1b; **(e)** Presence of clinical symptoms; **(f)** Necrosis on imaging; Abbreviations: OR, odds ratio; CI, confidence interval; OR, odds ratio; BMI, body mass index; pPruthi, 2023, patients in Pruthi, 2023 article treated with partial nephrectomy ;rPruthi, 2023, patients in Pruthi, 2023 article treated with radical nephrectomy.

**Figure 3:**
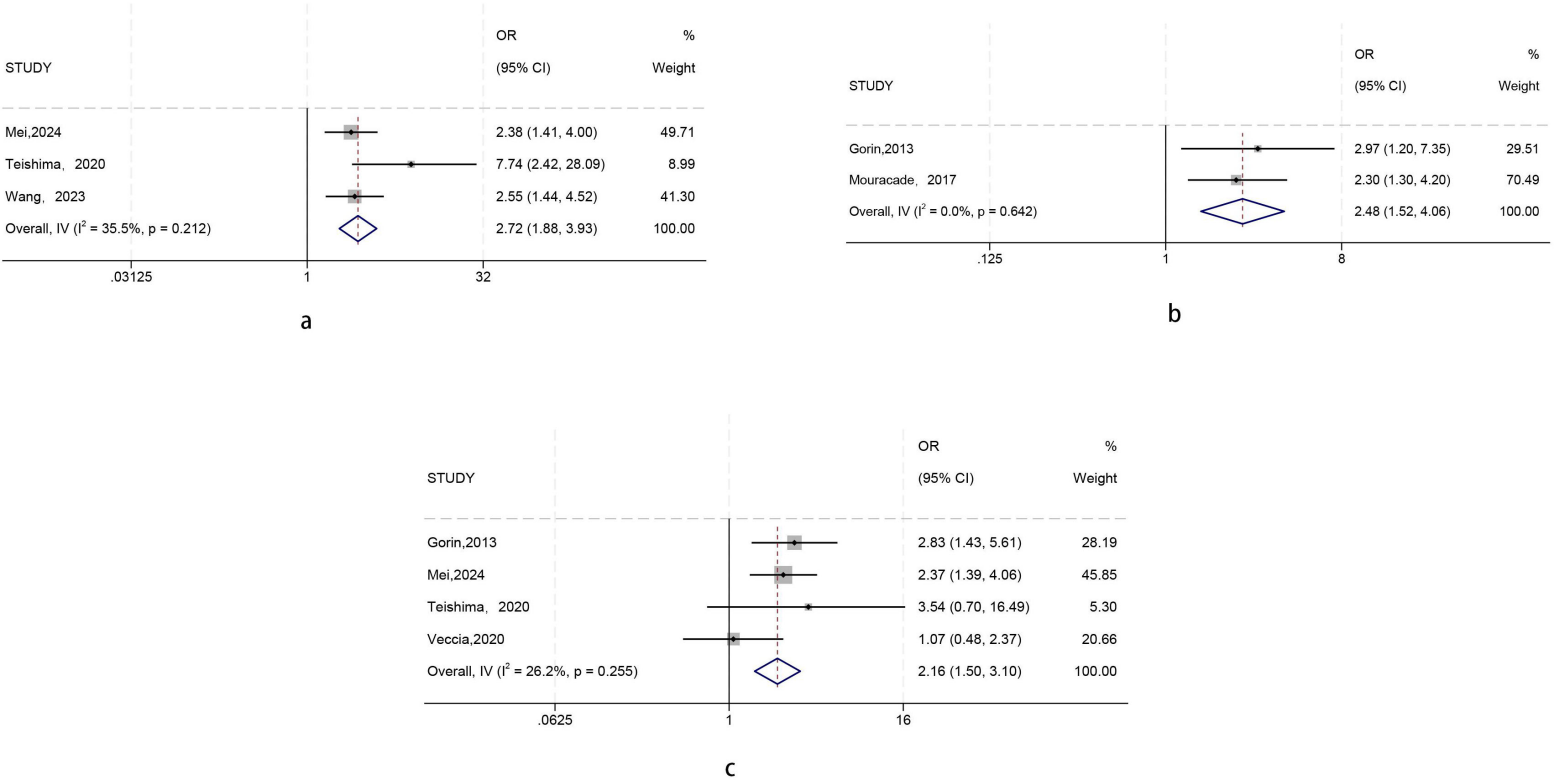
Forest plots of predictors of pathological upstaging. [**(a)** Irregular tumor margin; **(b)** R.E.N.A.L. score (score7-9); **(c)** renal hilum].

**Figure 4:**
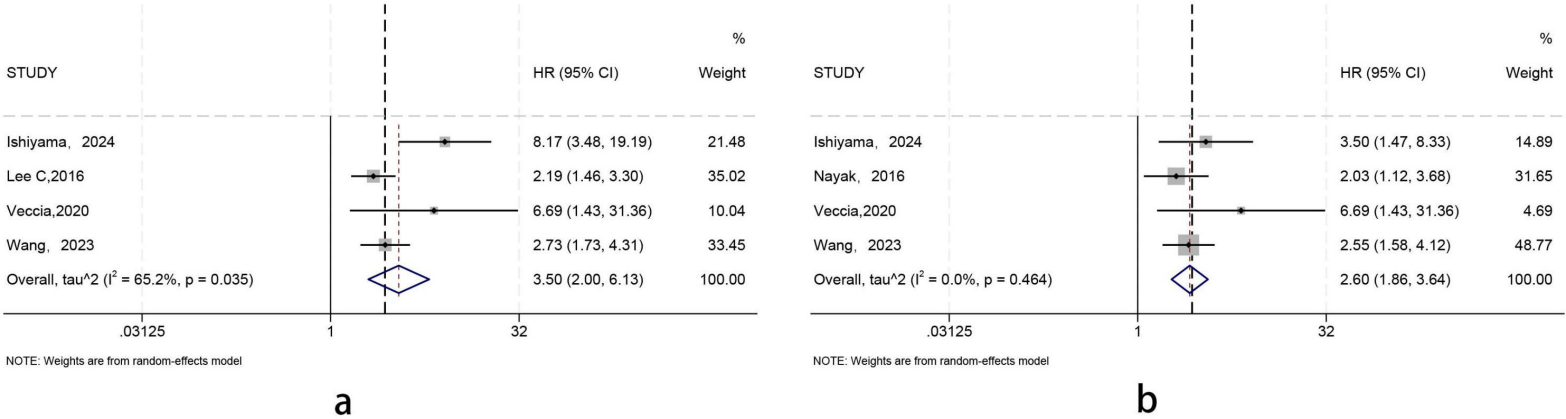
Forest plots of unadjusted and adjusted HRs for RFS in pT3a upstaging. [**(a)** the Unadjusted hazard ratios; **(b)** the Multivariate-adjusted hazard ratios].

Compared with the postoperative pT1 group, patients in the pT3 group were older (OR = 1.029; 95% CI= 1.02–1.038;p = 0.0001), had a higher proportion of males (OR = 1.067; 95% CI= 1.029–1.107;p= 0.001), and also have a higher BMI(Body Mass Index) (OR= 1.016;95% CI= 1.004–1.027;p= 0.0001) (**Figures 2**–**4**).

In the analysis of tumor size on imaging, we found a high level of heterogeneity (I² = 94.2%, p = 0.0001). Both the funnel plot and Egger’s plot also indicated high heterogeneity among the studies. We then conducted subgroup analyses based on ethnicity (Asian/non-Asian), study methodology (prospective study/retrospective study), and surgical approach (PN/combination of PN and RN), but the heterogeneity remained high. Therefore, tumor size was not included as a predictor. Interestingly, however, we found that patients with cT1b (OR= 5.865; 95% CI= 3.711- 9.267; p = 0.0001) were more likely to experience pathological upstaging to T3a after surgery (**Figure 2**).

In addition, patients presenting with clinical symptoms(OR= 1.987; 95% CI= 1.439- 2.744; p = 0.0001), the RENAL score of 7–9 (OR = 2.480; 95% CI= 1.516–4.058;p = 0.0001), necrosis on imaging(OR = 2.347;95% CI= 1.570–3.509;p = 0.0001),irregular tumor margins on imaging(OR = 2.874; 95% CI= 1.760–4.693;p = 0.0001) and renal hilum invasion (OR = 2.134;95%CI= 1.367-3.333;p = 0.001)appeared to be more prone to pathological upstaging to T3a after surgery (**Figures 2**, **3**).

We conducted a random-effects meta-analysis of the unadjusted hazard ratios (uHR) and multivariable-adjusted hazard ratios (aHR) from individual studies. The analysis revealed that postoperative pathological stage upstaging to pT3a was significantly associated with poorer recurrence-free survival (RFS) in patients with cT1 renal cell carcinoma. The unadjusted meta-analysis results indicated an increased risk of recurrence in patients who upstaged to pT3a (HR= 3.50,95% CI= 2.00-6.13), but moderate heterogeneity was observed between studies (I²=65.2%, Q-test p=0.035). After multivariable adjustment, the association remained consistent and in the same direction (HR= 2.60,95% CI= 1.86-3.64), with no significant heterogeneity between studies (I²=0%, Q-test p= 0.464) (**Figure 4**).

## 4 Discussion

This analysis included a total of approximately 24,957 patients, with cases of pathological upstaging accounting for 7.46% of the total study population. It can be seen that the proportion of cT1 RCC patients who experience pathological upstaging to pT3a after surgery is extremely small.

Advanced age has been recognized as an independent risk factor for postoperative pathological upstaging in RCC, and this association has been confirmed in most relevant studies (6, 14), possibly because older patients are more likely to harbor biologically aggressive tumors (6). Male patients constitute a fundamental risk factor. Most studies have identified gender as a risk factor, which may be attributed to the higher prevalence of perirenal fat adhesion in elderly and male patients (16). However, perirenal fat may be poorly visualized on imaging examinations, resulting in understaging before surgery. Moreover, we found that the presence of clinical symptoms was an independent predictor of upstaging and the proportion of symptomatic patients in the upstaging group was significantly higher. Given that the classic triad of flank pain, palpable abdominal mass, and gross hematuria is rare and associated with advanced disease and aggressive histology of RCC (2), attention should be paid to the risk of upstaging when RCC patients present with clinical symptoms.

In the study by Pruthi et al., emphasis was placed on describing the relationship between obesity and RCC. In our analysis, BMI also showed statistical significance. They proposed that clear cell renal cell carcinoma (ccRCC) develops in the proximal convoluted tubules of the renal cortex, which is also a common site of metabolic overload and injury induced by obesity (24). In addition, they emphasized the significance of diabetes in pathological upstaging. However, our study did not find this factor to be statistically significant. This is primarily due to the paucity of studies that have identified diabetes as a meaningful predictive factor in multivariate analyses. Nevertheless, metabolic factors play a relatively important role in tumor invasion and even tumor prognosis (25).

It should be noted that continuous tumor size was excluded due to high heterogeneity across studies, whereas cT1b stage was retained as a standardized clinical staging category with consistent definition, thereby resolving the apparent conceptual inconsistency. Clinically, cT1b is defined as tumors with a maximum diameter of 4–7 cm, which also represents a subgroup with a relatively larger maximum diameter within the clinical T1 stage. From an imaging perspective, nearly all studies have confirmed that tumor size is one of the independent predictive factors for pathological upstaging (12, 13, 26, 32). Specifically, increased tumor volume have been validated as important risk factors for pathological upstaging in multiple studies (8, 31). In Veccia’s study (30), tumors located in the renal hilum appeared more likely to be associated with pathological upstaging in the pathological upstaging group, but this association did not reach the conventional level of significance (p = 0.07). In contrast, the current study confirmed the role of hilar-located tumors as a predictive factor for pathological upstaging. A study conducted by Venkatesh et al. confirmed that patients with mesophytic renal masses (12.8%) had a significantly lower complication rate compared to those with hilar (50%) renal masses (31), which suggests a potential association between tumor location and tumor aggressiveness.

Irregular renal tumor margins have been identified as a strong predictive factor for perirenal/renal sinus fat invasion, postoperative pathological upstaging to pT3a, and aggressive histological subtypes (7, 11). In the study by Wang et al., irregular renal tumor margins were categorized into three types: nodular growth pattern, blurred boundary between the renal tumor and parenchyma, and completely non-elliptical morphology. This study confirmed that irregular renal tumor margins are an important independent risk factor for aggressive pathology in cT1-stage solid RCC. Studies by Collins Chen et al. revealed that pathological tumor necrosis is an independent prognostic factor for RCC patients. Additionally, tumors with necrosis have a higher probability of invading the collecting system, leading to poor prognosis. A report by U. Patel and H. Sokhi indicated that the presence of tumor necrosis, irregular tumor margins, or tumor invasion of the renal sinus or perirenal fat on computed tomography (CT) images increases the likelihood of local invasion (23). Consequently, there may be invasive components that cannot be detected by imaging, leading to postoperative pathological upstaging (1).

Notably, substantial heterogeneity was observed across included studies regarding imaging-derived predictors. These features generally lack standardized definitions, and variability exists in imaging modalities, acquisition parameters, radiologist experience, and reporting criteria. Beyond heterogeneity in tumor size, these methodological differences may compromise the reproducibility and external validity of imaging-based evidence. The invasion of perirenal fat by tumors imposes extremely high requirements on the sensitivity and accuracy of imaging modalities. Although imaging cannot reliably identify perirenal fat invasion, pathological examination can confirm the diagnosis postoperatively, thereby affecting preoperative and postoperative pathological staging. Therefore, imaging predictors should be considered as associated factors for pathological upstaging in clinical practice.

Beyond the aforementioned factors, our study also found that the RENAL score of 7–9 was statistically significant. In other studies, researchers generally agree that the high RENAL score (10–12, 20) is more likely to be associated with postoperative pathological upstaging. However, in our analysis of high RENAL scores, we observed that the OR data from Ishiyama et al. differed from those of three other studies (Gorin et al., Mouracade et al., and Pathak et al.). Specifically, in Ishiyama et al.’s study, the proportion of patients with a high RENAL score in the pathological upstaging group was smaller than that in the non-upstaging group, which was contrary to the findings of the other three studies. This discrepancy prevented us from obtaining a statistically significant result for high RENAL scores in the meta-analysis. Therefore, we could not confirm high RENAL score as a significant predictive factor; only a RENAL score of 7–9 remained statistically significant in our analysis.

In the analysis, we conducted a meta-analysis of random-effects models using unadjusted hazard ratios (uHR) and multivariable adjusted hazard ratios (aHR). The meta-analysis demonstrated a significant association between postoperative pathological stage upstaging to pT3a and poorer recurrence-free survival (RFS) in patients with cT1 renal cell carcinoma. This finding was also corroborated by multiple studies (30, 33). Therefore, when confirming a low tumor stage, greater consideration should be given to the possibility of an increase in the actual pathological stage. This enables the adoption of more effective treatment approaches and avoids poorer treatment outcomes.

Compared with previous studies, our article has incorporated some new data. In previous articles, positive surgical margins (PSM) were also considered as a predictive factor. However, we believe that PSM is a factor that cannot be estimated preoperatively, so we have excluded it from our consideration.

This study has several limitations. First, most included studies were retrospective in design. Although the Newcastle-Ottawa Scale was used for quality assessment, the risk of selection bias, information bias, and confounding bias remains a concern due to incomplete historical records, variations in case selection criteria, and insufficient control of confounding factors. Accordingly, study quality and potential risk of bias may have influenced the overall results, which could compromise the accuracy of the data and the reliability of the conclusions. Second, the variability in clinical treatment decisions represented a significant source of confounding. Individualized differences among medical teams regarding surgical timing, adjuvant treatment regimens, and perioperative management could interfere with the objectivity of pathological assessment, leading to inconsistencies in the study conclusions. Notably, the choice of surgical approach may introduce bias; unlike radical nephrectomy, partial nephrectomy does not involve the systematic dissection of the main renal vein, potentially leading to an underestimation of venous invasion and subsequent pathological upstaging. Third, this study did not account for differences in imaging technologies across different years and regions. Such discrepancies may have caused biases in the evaluation of key indicators (e.g., tumor size, invasive extent), thereby affecting the accuracy of the results. Fourth, the pathological diagnostic criteria for pT3a in the study were not standardized. Variations in the pathological assessment standards for perirenal fat invasion, renal sinus involvement, and venous invasion across different centers and time periods may substantially affect outcome classification, thereby potentially interfering with the accuracy of predictive factor association analyses with pT3a staging. Fifth, another significant limitation of this study is the exclusion of tumor biological characteristics. Due to the study’s constraints, this meta-analysis focused solely on clinical and imaging predictors, without analyzing molecular, genomic, or histological markers associated with tumor invasive behavior. Existing studies have demonstrated that biological features such as elevated Ki-67 proliferation index (15) are closely related to the invasiveness of renal cell carcinoma and may contribute to the development of occult pT3a. However, this study did not incorporate these features into the analysis, making it impossible to definitively determine the supplementary role of such biological indicators in clinical predictors. Although heterogeneity was formally evaluated in the present meta-analysis, the included studies still exhibited heterogeneity with respect to imaging protocols, surgical approaches, pathological definitions of pT3a, and follow-up strategies. Accordingly, the clinical significance of some pooled odds ratios, especially those with small effect sizes including age, BMI, and sex, should be interpreted with caution. These limitations should be taken into consideration when applying the pooled results to clinical practice. Additionally, some predictive factors were only investigated in a small number of studies with limited sample sizes. Whether these factors can be recognized as independent predictive factors requires further validation in large-sample, high-quality studies.

## 5 Conclusion

Current evidence suggests that nine variables, including advanced age, gender, and irregular margins, may be predictive of postoperative pathological upstaging in patients with cT1 renal cell carcinoma. The present study validates and consolidates these previously established risk factors rather than identifying novel predictors. In clinical practice, individualized risk assessment may be further optimized using these well−supported clinicoradiological factors.

## Data Availability

The original contributions presented in the study are included in the article/supplementary material. Further inquiries can be directed to the corresponding authors.
